# m^6^A reader hnRNPA2B1 drives multiple myeloma osteolytic bone disease

**DOI:** 10.7150/thno.76852

**Published:** 2022-11-14

**Authors:** Rui Liu, Yuping Zhong, Rui Chen, Chengchao Chu, Gang Liu, Yong Zhou, Yazhu Huang, Zhihong Fang, Huan Liu

**Affiliations:** 1 Cancer Research Center, School of Medicine, Xiamen University, Xiamen, 361102, China.; 2 Department of Hematology, Qingdao Municipal Hospital, School of Medicine, Qingdao University, Qingdao, 266011, China.; 3 Eye Institute of Xiamen University, Fujian Provincial Key Laboratory of Ophthalmology and Visual Science, School of Medicine, Xiamen University, Xiamen 361102, China.; 4 State Key Laboratory of Molecular Vaccinology and Molecular Diagnostics & Center for Molecular Imaging and Translational Medicine, School of Public Health, Xiamen University, Xiamen 361102, China.; 5 Department of Hematology, The First Affiliated Hospital of Xiamen University and Institute of Hematology, School of Medicine, Xiamen University, Xiamen, 361102, China.; 6 Department of Hematology, Key Laboratory of Xiamen for Diagnosis and Treatment of Hematological Malignancy, Xiamen, 361102, China.; 7 Fujian Provincial Key Laboratory of Organ and Tissue Regeneration, Xiamen Key Laboratory of Regeneration Medicine, Organ Transplantation Institute of Xiamen University, School of Medicine, Xiamen University, Xiamen, 361102, China.; 8 Shenzhen Research Institute of Xiamen University, Shenzhen, Guangdong 518057, China.

**Keywords:** Multiple myeloma, bone lesion, hnRNPA2B1, exosome, microRNA

## Abstract

**Rationale:** Bone destruction is a hallmark of multiple myeloma (MM) and affects more than 80% of patients. Although previous works revealed the roles of N^6^-methyladenosine (m6A) reader hnRNPA2B1 in the development of tumors, whether hnRNPA2B1 regulates bone destruction in MM is still unknown.

**Methods:** Alizarin red S staining, TRAP staining, ELISA and quantitative real-time PCR assays were used to evaluate osteogenesis and osteoclastogenesis *in vitro*. X ray and bone histomorphometric analysis were preformed to identify bone resorption and bone formation *in vivo*. Exosome isolation and characterization were demonstrated by transmission electron microscopy, dynamic light scattering, immunofluorescence and flow cytometry assays. The interactions between hnRNPA2B1 and primary microRNAs were examined using RNA pull-down and RIP assays. Coimmunoprecipitation assay was used to test the interaction between hnRNPA2B1 and DGCR8 proteins. Luciferase assay was established to assess miRNAs target genes.

**Results:** Here we show that myeloma cells hnRNPA2B1 mediates microRNAs processing and upregulates miR-92a-2-5p and miR-373-3p expression. These two microRNAs are transported to recipient monocytes or mesenchymal stem cells (MSCs) through exosomes, leading to activation of osteoclastogenesis and suppression of osteoblastogenesis by inhibiting IRF8 or RUNX2. Furthermore, clinical studies revealed a highly positive correlation between the level of myeloma cells hnRNPA2B1 and the number of osteolytic bone lesions in myeloma patients.

**Conclusions:** This study elucidates an important mechanism by which myeloma-induced bone lesions, suggesting that hnRNPA2B1 may be targeted to prevent myeloma-associated bone disease.

## Introduction

Multiple myeloma (MM) arises from malignant plasma cells within bone marrow and remains an incurable disease. One hallmark of MM is osteolytic bone disease. More than 80% of patients suffer from bone destruction, which includes pathological fracture, severe bone pain, spinal cord compression, and hypercalcemia, greatly reduces their quality of life and has a severe negative impact on survival 1.

Bone is a dynamic tissue that is constantly being remodeled by bone-resorbing osteoclasts and bone-forming osteoblasts 2. Osteoclasts arise from hematopoietic monocytic precursors and resorbs bone. The formation of osteoclasts requires soluble cytokines such as receptor activator of nuclear factor-κB ligand (RANKL) and macrophage colony-stimulating factor (M-CSF). RANKL enhances the expression of nuclear factor of activated T-cells, cytoplasmic 1 protein (NFATc1), which upregulates the expression of osteoclast differentiation-associated genes, such as tartrate-resistant acid phosphatase (*TRAP*), calcitonin receptor (*CALCR*), and cathepsin K (*CTSK*), whereas the transcriptional factor interferon regulatory factor 8 (IRF8) can suppress RANKL-induced NFATc1 expression 2. The next player in the remodeling cycle is the osteoblasts, which are differentiated from mesenchymal stem cells (MSCs). Osteoblast's differentiation requires the activation of core-binding factor α-1/runt-related transcription factor 2 (*RUNX2*) and osterix, which stimulate the expression of osteoblast differentiation-associated genes, such as bone gamma-carboxyglutamic acid-containing protein (*BGLAP*), alkaline phosphatase (*ALP*), and collagen type I α1 (*COL1A1*) 2, 3. This delicate balance is disrupted in certain types of malignancies, including MM and solid tumors, such as breast and lung cancer 4, 5. Myeloma cells can stimulate production of several cytokines such as RANKL, macrophage inflammatory protein-1α (MIP-1α), and monocyte chemoattractant protein-1 (MCP-1) and thus enhance osteoclast differentiation and bone resorption activity 4, 6. On the other hand, myeloma cells can also secrete dickkopf-related protein 1 (DKK1), which inhibits the Wnt/β-catenin signaling pathway and suppresses maturation of MSCs into osteoblasts 7.

N6-methyladenosine (m6A) modification is the most abundant modification on eukaryotic RNA. The modification is composed of three classes of protein factors: methylate adenosine at N6 position (writers), demethylate m6A for reversible regulation (erasers) and effectors that recognize and bind to m6A motif, regulating RNA stability, splicing, trafficking, and mRNA translation and others (Readers) 8. The heterogeneous nuclear ribonucleoprotein A2B1 (hnRNPA2B1) is one of the nuclear readers of m6A, which is highly expressed in many types of cancers and accelerate mRNA processing via RNA binding, indicating an important role in the development of tumors 9, 10. For instance, hnRNPA2B1 results in pyruvate kinase isozymes M2 (PKM2) cumulation and promotes glioma cells proliferation 11. In pancreatic ductal adenocarcinoma cells, hnRNPA2B1 interacts with Kirsten rat sarcoma viral oncogene (KRAS) and modulated cell proliferation, mobility, and apoptosis 12. During epithelial mesenchymal transition (EMT) in tumor cells, hnRNPA2B1 up-regulates vimentin and N-cadherin and down-regulates E-cadherin, promotes cell invasion and metastasis in various cancers 13. In MM, recent work reveals m6A-dependent effect of hnRNPA2B1 on activating AKT signaling pathway and promoting MM progression 14. However, hnRNPA2B1 has never been implicated in the regulation of bone resorption or formation in tumors. In this work, we hypothesized that the hnRNPA2B1 plays a role in the pathogenesis of cancer-induced bone destruction in myeloma.

Through a combination of *in vitro*, *in vivo*, and patient samples study, we reported that hnRNPA2B1 has a unique role in myeloma-induced bone disease. Our results showed that myeloma cell hnRNPA2B1-DGCR8 (DiGeorge syndrome critical region 8) complex upregulates miR-92a-2-5p and miR-373-3p in myeloma cell exosomes. Exosomes miR-92a-2-5p inhibits the expression of interferon regulatory factor 8 (IRF8) and thereby activates RANKL-induced NFATc1 expression, leading to an increase in osteoclastogenesis and bone resorption. Exosomes miR-373-3p suppresses osteoblastogenesis and bone formation by downregulating the expression of RUNX2 in human MSCs. Our findings not only elucidate a mechanism of cancer-induced bone destruction, but also implicate a potential therapeutic approach for cancer patients with osteolytic bone lesions by targeting hnRNPA2B1.

## Results

### hnRNPA2B1 enhances lytic bone lesions and tumor progression

**Figure 1:**
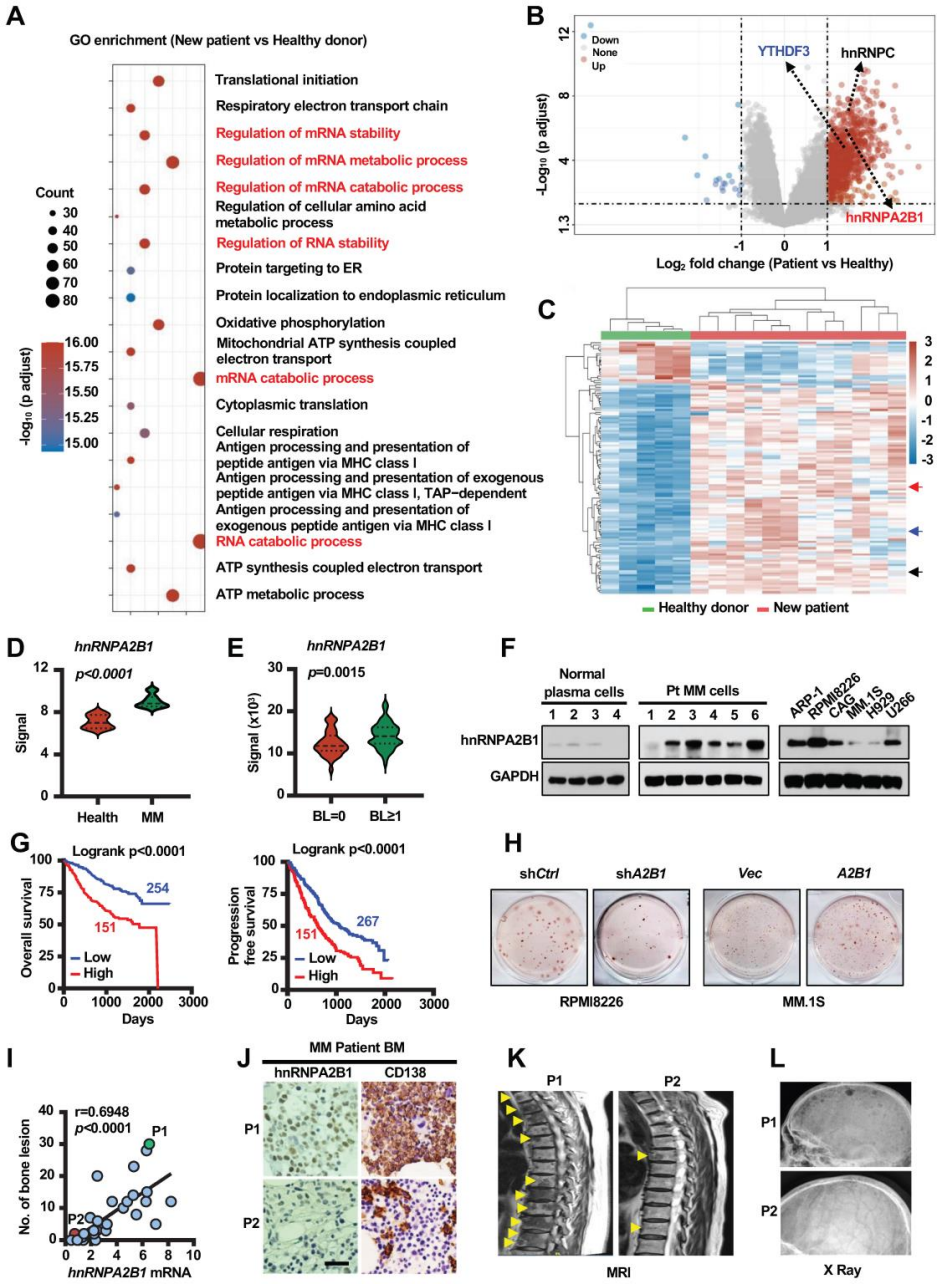
** hnRNPA2B1 is critical for myeloma disease progression. (A)** Pathway enrichment analysis in the plasma cells from myeloma patients (n = 12) compared to normal plasma cells from healthy donors (n = 5) (GEO: GSE6691). **(B)** Volcano plot showing genes with a cutoff fold-change of ≥ 2 or ≤ -2 and *P* values of <0.05. **(C)** Heatmap showing the expression profile of genes upregulated or downregulated. **(D)** *hnRNPA2B1* mRNA levels in GSE6691. **(E)** *hnRNPA2B1* mRNA levels in malignant plasma cells of 37 myeloma patients without bone lesion (BL = 0) and 136 myeloma patients with bone lesion (BL ≥ 1) (GEO: GSE755). Data shown as average ± SD. *P* values were determined by Student's *t* test.** (F)** Western blot analysis of hnRNPA2B1 expression in normal plasma cells, malignant plasma cells (Pt), and human myeloma cell lines. GAPDH served as loading control. Data are representative of triplicate blots. **(G)** Overall survival (OS) and Progression free survival (PFS) of myeloma patients with high hnRNPA2B1 (High) or low hnRNPA2B1(Low) expression.** (H)** Myeloma cell line RPMI8226 was infected with lentivirus carrying non-targeted control (sh*Ctrl*) or short-hairpin RNAs against human hnRNPA2B1 (sh*A2B1*). MM.1S was infected with lentivirus carrying control vector (*Vec*) or *hnRNPA2B1* complementary DNA (*A2B1*). After 3 weeks of culture in soft agar, the colonies were stained and visualized. Shown are representative images of sh*A2B1* (left panel) or *A2B1* (right panel) myeloma cell colonies. Each experiment was repeated three times. **(I)** Correlation coefficient of the mRNA levels of *hnRNPA2B1* and numbers of bone lesion in myeloma patients (n = 30). The correlations were evaluated using Pearson coefficient. r, correlation coefficient. *P* value was determined by Pearson coefficient. **(J)** Representative images of immunohistochemical staining show the expression of CD138 and hnRNPA2B1 in the biopsy segment of two patients (P1 and P2) which are highlighted with green or red color. Scale bar, 50 µm. Representative images of magnetic resonance imaging and X ray scanning for lytic lesions in the spine** (K)** and skull **(L)** in P1 and P2. Yellow arrows, bone lesion.

We analyzed the alteration of gene expression in plasma cells of myeloma patients, compared to plasma cells of healthy donors, using GEO dataset (GSE6691) 15. Computational overlapping of genes with the Molecular Signatures Database (Broad Institute) hallmark gene sets suggested significant enrichment of genes in mRNA stability and metabolic process (**Figure 1A**). As m6A modification proteins (METTL3, WTAP, FTO, hnRNPC, hnRNPA2B1, YTHDF3) are well known regulators on eukaryotic RNA processing. Paired differential analysis identified three of them (*hnRNPA2B1*, *YTHDF3* and *hnRNPC*) are significantly upregulated in plasma cells of myeloma patients compared to normal plasma cells (Fold Change > 2) (**Figure 1B**). Unsupervised hierarchical clustering of microarray data also suggested that these three genes expression were increased in the plasma cells of myeloma patients (**Figure 1C-D** and **Figure S1A**). Additionally, to understand whether m6A modification proteins may also affect myeloma cell-induced bone lesions, we compared their expression levels in myeloma cells of patients with or without bone lesions from a published dataset (GEO: GSE755) 7. Among the three candidate genes, the levels of *hnRNPA2B1* expression were higher in myeloma cells of patients with bone lesions compared with those without (**Figure 1E** and **Figure S1B**). Western blot results also showed that hnRNPA2B1 was expressed in most of the bone marrow aspirates of primary myeloma cells and in most of the established human myeloma cell lines, but not in aspirates of plasma cells from normal subjects (**Figure 1F**). We next analyzed the association of hnRNPA2B1 and other m6A modification proteins with myeloma disease using dataset from Multiple Myeloma Research Foundation (MMRF) coMMpass study IA15, and found that patients with high levels of hnRNPA2B1 in myeloma cells displayed shorter overall survival or progression free survival than those with low expression (**Figure 1G** and **Figure S1C**). Based on these results, hnRNPA2B1 was selected as candidate gene, which is highly likely a proliferation-related and bone lesion-related gene in myeloma.

To examine the functional role of hnRNPA2B1 in tumor growth and bone lesions, we knocked down its expression in RPMI8226 myeloma cells using small hairpin RNAs (shRNAs) against human *hnRNPA2B1* and over expressed *hnRNPA2B1* complementary DNA (cDNA; *A2B1*) in MM.1S myeloma cells (**Figure S2A**). We observed decreased colony formation and growth in sh*A2B1* myeloma cells compared with sh*Ctrl* cells (**Figure 1H** and **Figure S2B-G**), and increased colony formation and growth in *A2B1* myeloma cells compared with *Vec* cells (**Figure 1H** and **Figure S2B-G**). Furthermore, we found a strong positive correlation between the level of *hnRNPA2B1*expression in myeloma cells and bone lesion numbers in patients (**Figure 1I**). hnRNPA2B1 and CD138 (myeloma cells surface marker) expression were higher in myeloma cells from patient with high bone lesion numbers (P1) than in those from patient with low lesion numbers (P2) (**Figure 1J**). Representative images of magnetic resonance imaging and X ray scanning showed more lytic lesions in the spine (**Figure 1K**) and skull (**Figure 1L**) of P1 than P2. These results indicate the association of hnRNPA2B1 to myeloma tumorigenesis and bone disease.

**Figure 2:**
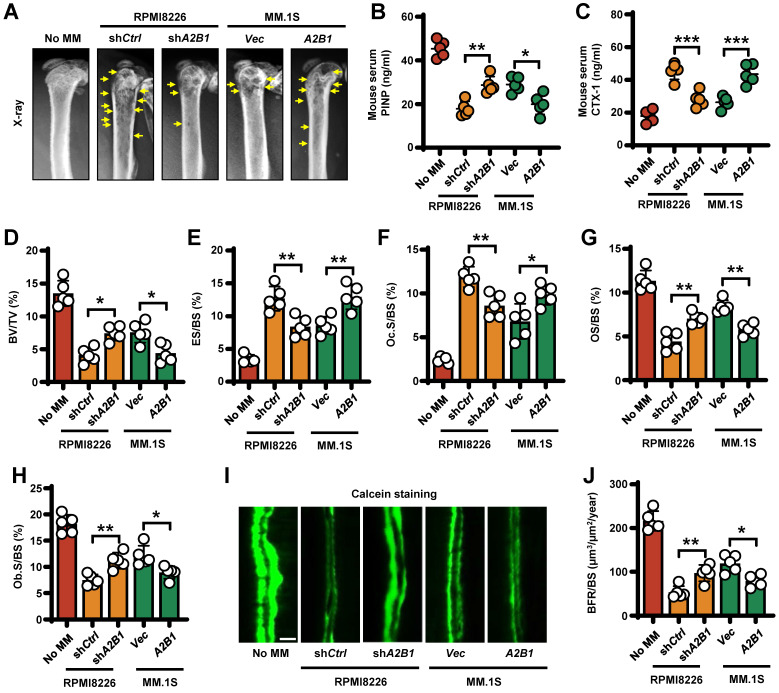
** Myeloma cells hnRNPA2B1 enhances bone resorption and inhibits bone formation *in vivo*.** SCID mice femurs injected with myeloma cell lines RPMI8226 [nontargeted shRNA (sh*Ctrl*), or *hnRNPA2B1* shRNA (sh*A2B1*)] or MM.1S [control vector (*Vec*), or *hnRNPA2B1* cDNA (*A2B1*)]. The mice not receiving myeloma cells (No MM) served as controls. Shown are the representative x-ray images of lytic lesions** (A)**, concentrations of PINP **(B)** or CTX-1 **(C)** in mouse sera, percentages of BV/TV** (D)**, ES/BS **(E)**, Oc.S/BS **(F)**, OS/BS **(G)**, and Ob.S/BS **(H)**. **(I, J)** Bone formation rate (BFR/BS) was measured by calcein injection, and the bone sections were imaged and analyzed. Shown are representative images and summarized data of bone formation in mouse femurs. Yellow arrows, bone lesion. Scale bar: 20 µm. Data are means ± SD (n = 5 mice/group, three replicate studies). *P < 0.05; **P < 0.01; ***P < 0.001. All *P* values were determined using one-way ANOVA.

To determine the functional role of myeloma expressed hnRNPA2B1 in lytic bone lesions, we injected sh*A2B1* RPMI8226 cells into mouse femurs and caused fewer lytic lesions than did sh*Ctrl* RPMI8226 cells. Conversely, *A2B1* MM.1S cells caused more femur lesions than did *Vec* MM.1S cells (**Figure 2A**). To assess the role of myeloma-expressed hnRNPA2B1 in osteoclast-mediated bone resorption *in vivo*, we examined the levels of mouse serum procollagen type I N-terminal propeptide (PINP), a bone formation marker and C-telopeptide of type I collagen (CTX-1), a bone resorption marker. We found higher PINP levels and lower CTX-1 levels in sh*A2B1* or *Vec* group, compared with sh*Ctrl* or *A2B1* group (**Figure 2B-C**). We also stained myeloma-bearing mouse femurs for TRAP and Toluidine blue, bone histomorphometric analysis demonstrated a lower bone volume/total volume (BV/TV) (**Figure 2D**), higher percentage of bone surface eroded by osteoclasts (ES/BS) (**Figure 2E**), percentage of bone surface covered with osteoclasts (Oc. S/BS) (**Figure 2F**), and lower percentage of osteoid surface (OS/BS) (**Figure 2G**), bone surface lined with osteoblasts (Ob. S/BS) (**Figure 2H**) and bone formation rate (**Figure 2I-J**) in the mice injected with myeloma cells expressing high levels of hnRNPA2B1 (sh*Ctrl* or *A2B1*) than in those injected with low hnRNPA2B1 myeloma cells (sh*A2B1* or *Vec*). Together, these results reveal that myeloma cells express hnRNPA2B1 enhances lytic bone lesions and tumor progression in patients and mice model with myeloma.

### hnRNPA2B1 enhances RANKL-induced osteoclastogenesis and inhibits osteoblastogenesis through exosomes

**Figure 3:**
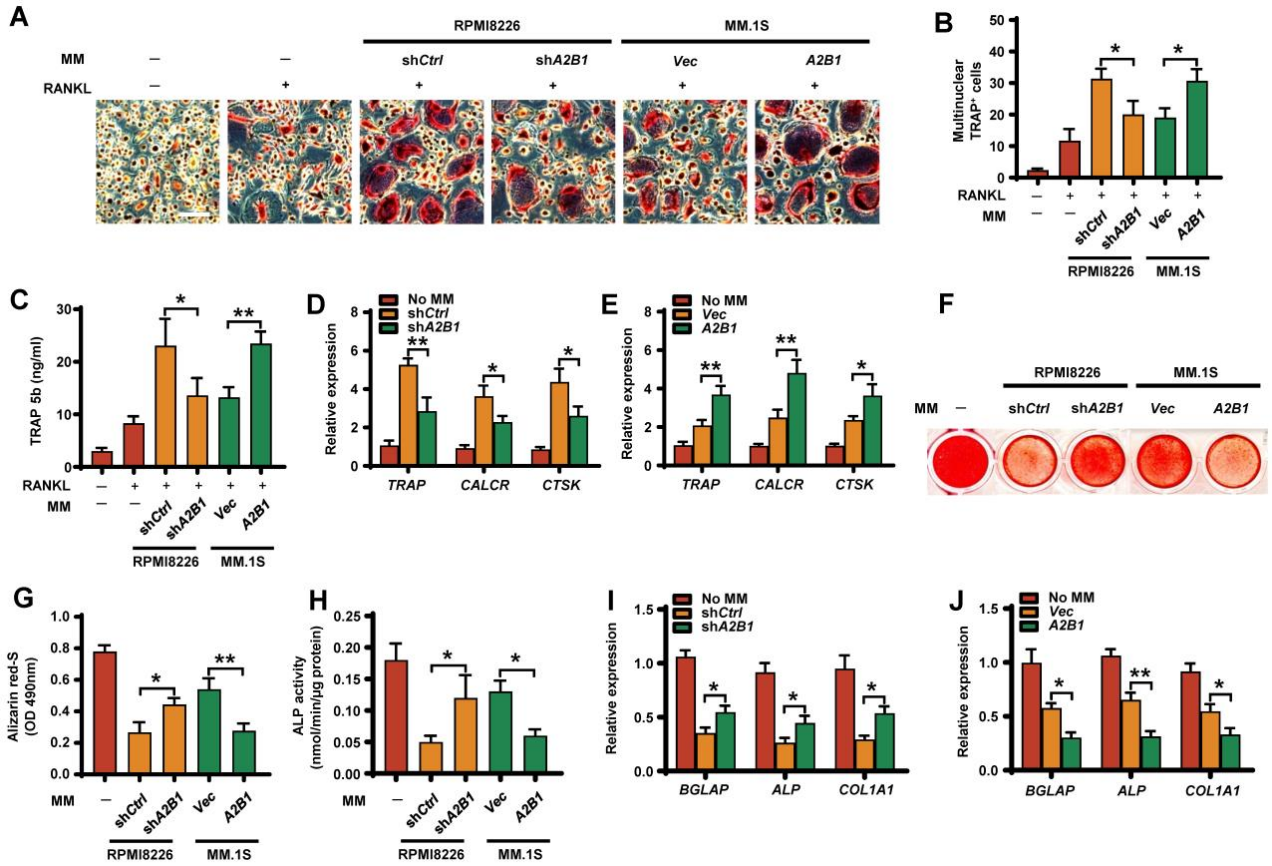
** Myeloma cells hnRNPA2B1 enhances osteoclast differentiation and inhibits osteoblast differentiation *in vitro*.** Precursors of osteoclasts were cocultured with myeloma cell lines RPMI8226 (sh*Ctrl*, sh*A2B1*) or MM.1S (*Vec*, *A2B1*) in osteoclast medium. Shown are the numbers of multinuclear (≥ 3) TRAP^+^ cells **(A, B)**, TRAP 5b **(C)**, and relative expression of the *TRAP*, *CALCR*, and *CTSK* genes in precursors of osteoclasts **(D, E)**. Scale bars, 100 µm. MSCs were cocultured with myeloma cell lines RPMI8226 (sh*Ctrl*, sh*A2B1*) or MM.1S (*Vec*, *A2B1*) in osteoblast medium. Shown are representative images **(F)** and summarized data of Alizarin red S staining **(G)**, ALP activity **(H)**, and the relative expression of *BGLAP*, *ALP*, and *COL1A1* genes in MSCs **(I, J)**. Data are averages ± SD. Each experiment was repeated three times. *P < 0.05; **P < 0.01. All *P* values were determined using one-way ANOVA.

Bone remodeling is maintained by a balance between osteoclast-mediated resorption and osteoblast-mediated bone formation. To examine whether myeloma cells hnRNPA2B1 can regulate this balance, we first assessed their effects on osteoclast differentiation. In the presence of RANKL, coculture of precursors of osteoclasts (preOCs) with myeloma cells expressing high levels of hnRNPA2B1 (sh*Ctrl* RPMI8226 or *A2B1* MM.1S) induced more multinuclear tartrate-resistant acid phosphatase-positive (TRAP^+^) cells formation (**Figure 3A-B**), TRAP 5b secretion (**Figure 3C**), and osteoclast gene expression (**Figure 3D-E**) than in those cocultured with low hnRNPA2B1 myeloma cells (sh*A2B1* or *Vec*). These results indicate that myeloma cells hnRNPA2B1 enhances osteoclastogenesis.

To assess the effect of myeloma cells hnRNPA2B1 on osteoblast formation, we cocultured osteoblast progenitors, MSCs, in osteoblast medium with myeloma cells. MSCs cultured alone in this medium served as a positive control. Cocultured with low hnRNPA2B1-expressing myeloma cell lines (*Vec* MM.1S, or sh*A2B1* RPMI8226) had comparatively more mature osteoblasts (**Figure 3F-G**), higher ALP activities (**Figure 3H**), and higher expression of osteoblast differentiation-associated genes (**Figure 3I-J**) than those with high levels of hnRNPA2B1. These results indicate that myeloma-expressed hnRNPA2B1 inhibits osteoblastogenesis.

**Figure 4:**
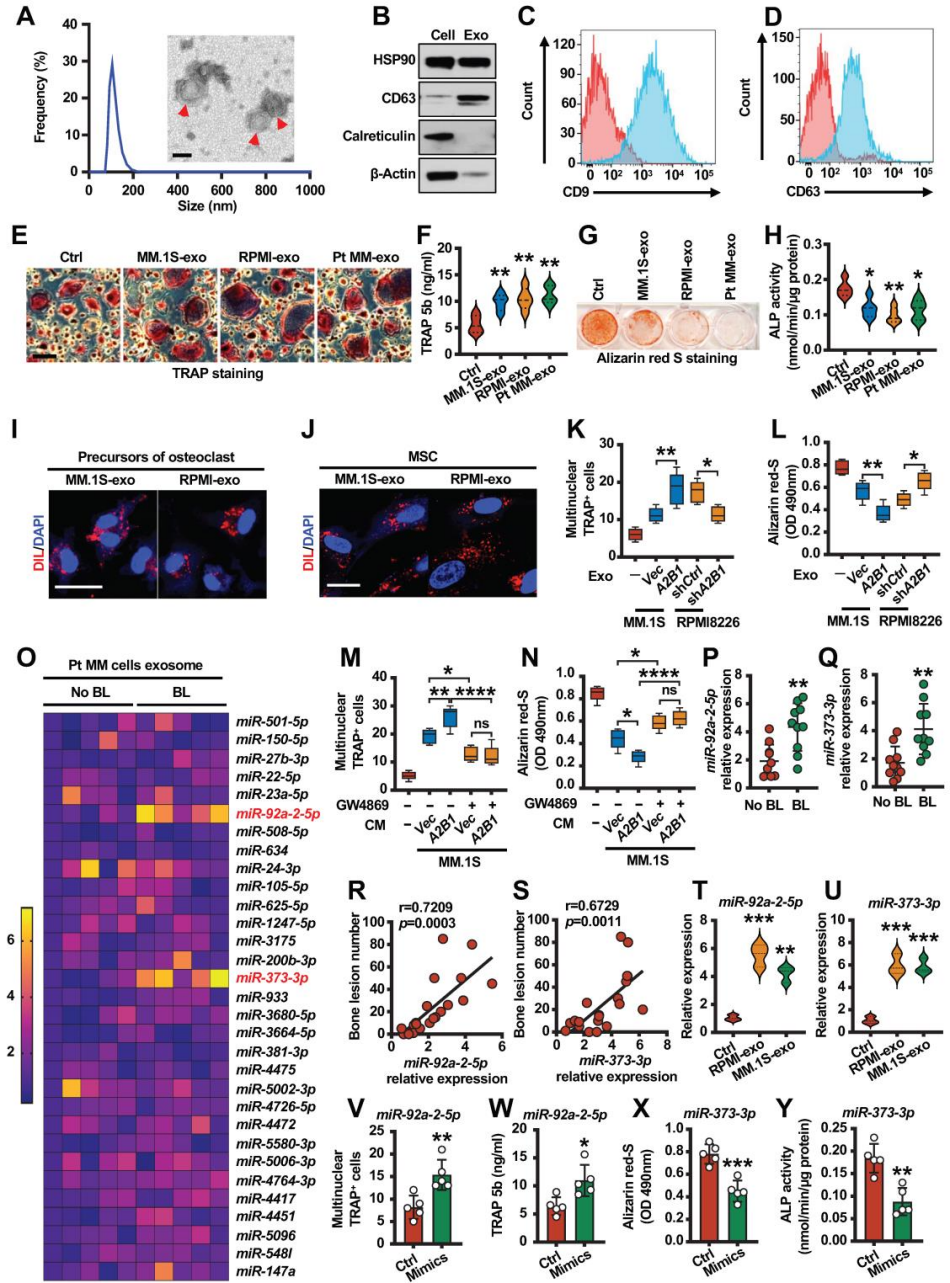
** miR-92a-2-5p and miR-373-3p are upregulated in myeloma cell exosomes and promotes bone destruction. (A)** Dynamic light scattering (DLS) results for the size distribution and electron microscopic image of exosomes derived from culture medium of patient myeloma cells. Scale bar, 100 nm. Red arrows: Exosome.** (B)** Western blot analysis for HSP90, CD63, Calreticulin and β-actin in patient myeloma cells and exosomes. **(C, D)** Specific surface marker proteins CD9 and CD63 of exosome detected by flow cytometry. Precursors of osteoclasts were cultured in osteoclast medium treated with exosomes (20 µg/ml) isolated from MM.1S culture medium (MM.1S-exo), RPMI8226 culture medium (RPMI-exo) or patient myeloma cells culture medium (Pt MM-exo). Shown are the morphologies of multinuclear (≥ 3) TRAP^+^ cells **(E)** and TRAP 5b levels in cell culture supernatant** (F)**. Scale bars, 100 µm. MSCs were cultured in osteoblast medium treated with MM.1S-exo, RPMI-exo or Pt MM-exo. Shown are representative images of Alizarin red S staining **(G)** and ALP activity **(H)** in MSCs. **(I, J)** Uptake of the red fluorescence dye Dil-labeled MM.1S-exo or RPMI-exo by precursors of osteoclasts or MSCs. Scale bar, 10 µm. Precursors of osteoclasts or MSCs were cultured in osteoclast medium or osteoblast medium treated with exosomes isolated from MM.1S (*Vec* and *A2B1*) or RPMI8226 (sh*Ctrl* and sh*A2B1*) culture medium. Shown are numbers of multinuclear (≥ 3) TRAP^+^ cells **(K)** and summarized data of Alizarin red S staining **(L)**. Addition of PBS served as a control.** (M, N)** MM.1S (*Vec* and *A2B1*) cells were cultured with or without GW4869 (20 µM) for 2 days, given fresh medium, and cultured for another 2 days, and then the conditioned medium (CM) was collected. Precursors of osteoclasts or MSCs were cultured with or without the MM.1S CM. After culturing, the cells were subjected to Alizarin red-S staining (M) or TRAP staining assay (N). ns, not significant. **(O)** Quantitative real-time PCR analysis shows the relative expression of miRNAs in patient myeloma cells with (n = 5) or without (n = 5) bone lesions. **(P, Q)** Quantitative real-time PCR analysis shows the relative expression of *miR-92a-2-5p* (P) and *miR-373-3p* (Q) in patient myeloma cells with (n = 10) or without (n = 10) bone lesions.** (R, S)** Correlation coefficient between the levels of *miR-92a-2-5p* (R) or *miR-373-3p* (S) and numbers of bone lesion in myeloma patients (n = 30). The correlations were evaluated using Pearson coefficient. r, correlation coefficient. *P* value was determined by Pearson coefficient. **(T, U)** Quantitative real-time PCR analysis shows the relative expression of *miR-92a-2-5p* (T) and *miR-373-3p* (U) in precursors of osteoclasts or MSCs treated with RPMI-exo or MM.1S-exo. **(V, W)** Shown are numbers of multinuclear (≥ 3) TRAP^+^ cells (V) and TRAP 5b (W) in precursors of osteoclasts infected with mimics of *miR-92a-2-5p*. **(X, Y)** Shown are summarized data of Alizarin red S staining (X) and ALP activity (Y) in MSCs infected with mimics of *miR-373-3p*. Data are averages ± SD. Each experiment was repeated three times. *P < 0.05; **P < 0.01; ***P < 0.001; ****P < 0.0001. *P* values of P, Q, V-Y were determined by Student's *t* test. *P* values of F, H, K, L, M, N, T and U were determined using one way ANOVA.

Exosomes are small membrane vesicles (30-150 nm) derived from the luminal membranes of multivesicular bodies. Exosomes mediate local and systemic cell communication during tumor growth and progression through the horizontal transfer of information, such as mRNAs, microRNAs (miRNAs) and proteins 16, 17. In myeloma, other studies indicated that myeloma-exosomes modulate osteoclast and osteoblast function and differentiation, but the mechanisms are still unknown 18, 19. We isolated exosomes from cell culture medium using ultracentrifugation and confirmed their identity by electron microscopy and dynamic light scattering analysis (**Figure 4A**). This was further confirmed by the expression of exosome markers (**Figure 4B-D**). Osteoclastogenesis and osteoblastogenesis assay indicated that myeloma cells exosomes promote osteoclast differentiation (**Figure 4E-F** and **Figure S3A-B**) and inhibit osteoblast differentiation (**Figure 4G-H** and **Figure S3C-D**). To investigate whether exosomes can be taken up by precursors of osteoclasts or MSCs, a Dil dye was used to label the exosomes that were then co-cultured with target cells. Confocal microscopy showed that Dil signals were detected in cytoplasm of precursors of osteoclasts or MSCs (**Figure 4I-J**). Moreover, exosomes isolated from myeloma cells expressing high levels of hnRNPA2B1 (sh*Ctrl* RPMI8226 or *A2B1* MM.1S) induced more TRAP^+^ cells formation (**Figure 4K**) and less mature osteoblasts than those with low levels of hnRNPA2B1 (**Figure 4L**). We pre-treated MM.1S cells (*Vec* and *A2B1*) with or without GW4869 (Inhibitor of exosome biogenesis/release), and collected the conditioned medium (CM). Precursors of osteoclasts or MSCs were cultured with or without CM. The results showed that GW4869 significantly reversed CM induced osteoclastogenesis or inhibited osteoblastogenesis, and there is no difference between *Vec* and *A2B1* groups pre-treated with GW4869 (**Figure 4M-N**). These experiments demonstrate that hnRNPA2B1 enhances osteoclastogenesis and inhibits osteoblastogenesis through exosomes.

### hnRNPA2B1 upregulates exosomes miR-92a-2-5p and miR-373-3p expression and correlates with patient's bone lesions

hnRNPA2B1 was identified as a mediator of m6A-dependent primary miRNAs processing events 10. Thus, we hypothesized that hnRNPA2B1 regulates exosome miRNAs expression to induce bone lesions. We then divided myeloma patients into two groups on whether they suffered from bone lesions and cultured them to isolate exosomes. Using real time PCR analysis, we identified top two miRNAs: miR-92a-2-5p and miR-373-3p, which were upregulated in bone lesions group (**Figure 4O**). We confirmed the results by another 10 patient samples with bone lesions and 10 patient samples without bone lesions. The levels of miR-92a-2-5p and miR-373-3p expression were higher in myeloma cells of patients with bone lesions compared with those without (**Figure 4P-Q**). Furthermore, we found a strong positive correlation between the levels of miR-92a-2-5p or miR-373-3p in myeloma cells and bone lesion numbers in patients (**Figure 4R-S**). Real time PCR analysis indicated that miR-92a-2-5p or miR-373-3p upregulated in myeloma cell lines exosomes than healthy plasma cell exosomes (**Figure 4T-U**). To investigate the potential effect of miR-92a-2-5p on osteoclast differentiation, monocytes were transfected with miR-92a-2-5p mimics or control miRNA (**Figure S4A**). TRAP staining and TRAP 5b levels indicated that miR-92a-2-5p enhanced osteoclast differentiation (**Figure 4V-W**). In addition, MSCs were transfected with miR-373-3p mimics (**Figure S4B**). Alizarin red S staining and ALP activities experiments showed suppressed osteoblast differentiation (**Figure 4X-Y**).

**Figure 5:**
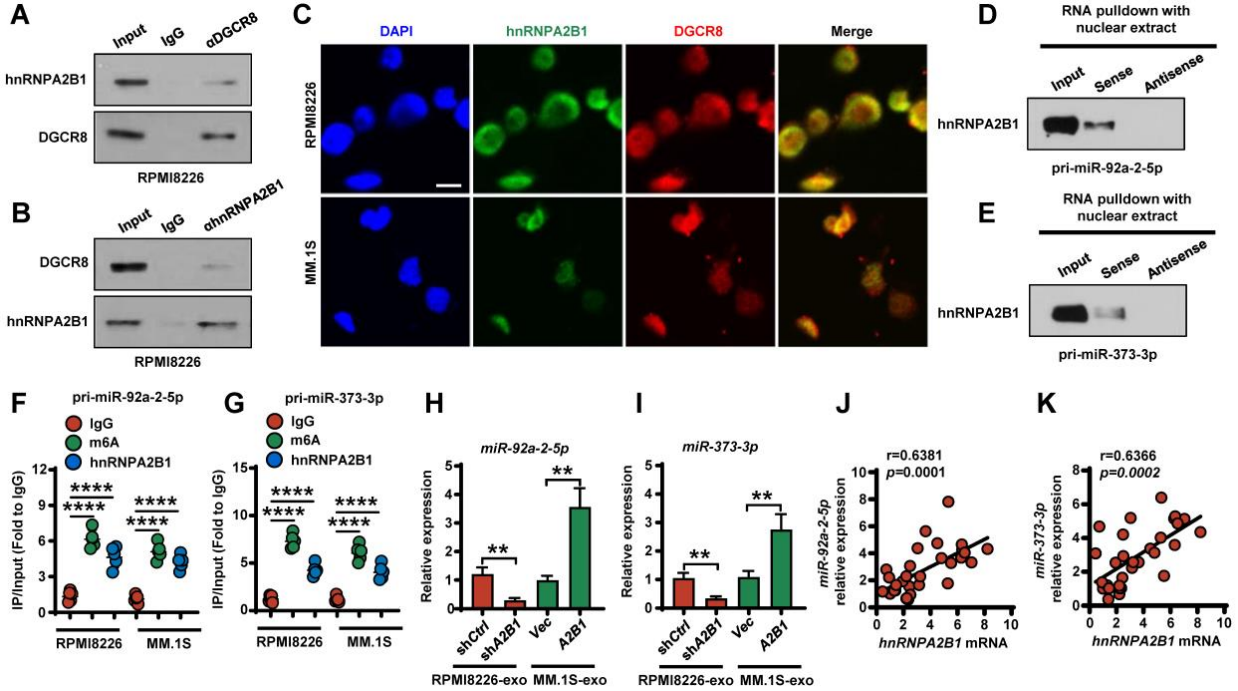
** hnRNPA2B1-DGCR8 complex binds to m6A pri-miRNA sequences and regulates miR-92a-2-5p and miR-373-3p processing. (A, B)** Coimmunoprecipitation of DGCR8 (A) or hnRNPA2B1 (B) in RPMI8226 cells. Data are representative of triplicate blots. **(C)** Immunofluorescent staining of RPMI8226 or MM.1S cells with DAPI and antibodies against hnRNPA2B1 or DGCR8. Scale bar, 5 μm. **(D, E)** RNA pull-down and Western blot with RPMI8226 cell nuclear extract confirmed binding between hnRNPA2B1 with primary miR-92a-2-5p (D) or primary miR-153-3p (E). **(F, G)** RIP analysis using the anti-hnRNPA2B1 or m6A antibody revealing that hnRNPA2B1 interacted with m6A-methylated primary miR-92a-2-5p (F) or primary miR-153-3p (G) in RPMI8226 or MM.1S cells. Negative control, IgG. **(H, I)** Quantitative real time PCR analysis shows the relative expression of *miR-92a-2-5p* (H) and *miR-373-3p* (I) in exosomes isolated from RPMI8226 (sh*Ctrl* and sh*A2B1*) or MM.1S (*Vec* and *A2B1*) culture medium. **(J, K)** Correlation coefficient between the levels of *miR-92a-2-5p* (J) or *miR-373-3p* (K) and *hnRNPA2B1* mRNA in patient myeloma cells (n = 30). The correlations were evaluated using Pearson coefficient. r, correlation coefficient. *P* values were determined by Pearson coefficient. Data are averages ± SD. Each experiment was repeated three times. **P < 0.01; ****P < 0.0001. *P* values were determined using one way ANOVA.

Next, we investigated the molecular mechanism of how hnRNPA2B1 regulates miR-92a-2-5p or miR-373-3p expression. Their expressions are higher in both the RPMI8226 and MM.1S cells and their corresponding exosomes as compared with that in monocytes or MSCs (**Figure S5A-B**). Previous study indicated that hnRNPA2B1 recruited microRNA microprocessor complex protein DGCR8 to a subset of precursor miRNAs and facilitates their processing into mature miRNAs 10. We used coimmunoprecipitation assays to interrogate the interaction between hnRNPA2B1 and DGCR8 proteins. We immunoprecipitated myeloma cells lysates with an anti-DGCR8 antibody and observed the presence of hnRNPA2B1 proteins in the lysates (**Figure 5A**). We also detected DGCR8 proteins in immunoprecipitates using an anti-hnRNPA2B1 antibody (**Figure 5B**), indicating that hnRNPA2B1 and DGCR8 form a complex. Using immunofluorescent staining, we observed a co-localization of hnRNPA2B1 and DGCR8 in myeloma cells (**Figure 5C**). Western blot analysis of enriched proteins after RNA pull-down indicated that primary miR-92a-2-5p or miR-373-3p bound specifically to hnRNPA2B1 (**Figure 5D-E**). RNA immunoprecipitation (RIP) showed enrichment of m6A-methylated primary miR-92a-2-5p or miR-373-3p by hnRNPA2B1 or m6A antibody, validating the interaction between primary miR-92a-2-5p or miR-373-3p and hnRNPA2B1 (**Figure 5F-G**). Knockdown of RPMI8226 hnRNPA2B1 reduced *miR-92a-2-5p* or *miR-373-3p* expression, and overexpressed of MM.1S hnRNPA2B1 increased *miR-92a-2-5p* or *miR-373-3p* expression (**Figure 5H-I**). In addition, we found a strong positive correlation between the levels of *miR-92a-2-5p* or *miR-373-3p* with *hnRNPA2B1* mRNA in patient myeloma cells (**Figure 5J-K**).

We further examined whether exosomal miR-92a-2-5p or miR-373-3p was successfully transferred into the monocytes or MSCs and found that miR-92a-2-5p or miR-373-3p expression was significantly increased in the monocytes or MSCs after incubation with the exosomes (**Figure S6A-B**). Furthermore, hnRNPA2B1 knockdown in RPMI8226 cells diminished recipient cells miR-92a-2-5p or miR-373-3p levels, whereas hnRNPA2B1 overexpressed in MM.1S cells increased miR-92a-2-5p or miR-373-3p levels in recipient cells (**Figure S6A-B**). Taken together, our results demonstrated that hnRNPA2B1-DGCR8 complex binds with primary miR-92a-2-5p or primary miR-373-3p and increased their mature miRNAs expression. Transported miR-92a-2-5p or miR-373-3p to recipient monocytes or MSCs induced more osteoclasts and less osteoblasts. These results indicate that hnRNPA2B1-DGCR8 complex upregulates exosomes miR-92a-2-5p and miR-373-3p expression, which activates osteoclastogenesis and osteoblastogenesis.

### hnRNPA2B1 regulated exosomes miR-92a-2-5p promotes NFATc1 expression and miR-373-3p inhibits RUNX2 expression

**Figure 6:**
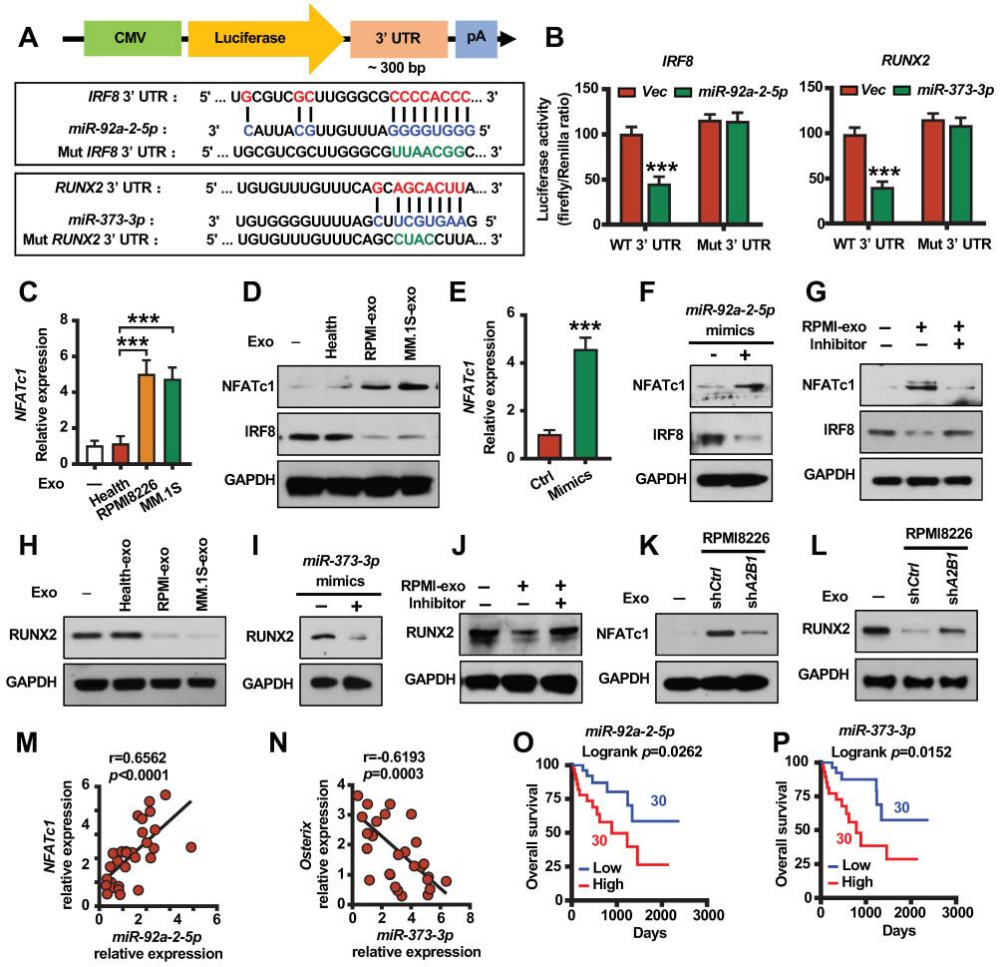
** hnRNPA2B1 regulated miR-92a-2-5p promotes NFATc1 expression and miR-373-3p inhibits RUNX2 expression. (A)** A diagram of *IRF8* and *RUNX2* 3' UTR reporters. (B) Luciferase readout from wide type or mutant *IRF8* or *RUNX2* 3' UTR reporter co-transfected in HEK293 cells with *miR-92a-2-5p*-expressing vector (left panel) or *miR-373-3p*-expressing vector (right panel) or empty vector (*Vec*). **(C)** Quantitative real-time PCR analysis shows the relative expression of *NFATc1* in precursors of osteoclasts treated with exosomes (20 µg/ml) isolated from healthy plasma cells (Health), RPMI8226 or MM.1S culture medium. **(D)** Western blot analysis of NFATc1 and IRF8 expression in precursors of osteoclasts treated with exosomes (Health-exo, RPMI-exo or MM.1S-exo). **(E)** Quantitative real-time PCR analysis shows the relative expression of *NFATc1* in precursors of osteoclasts infected with mimics of *miR-92a-2-5p*. *P* value was determined by Student's *t* test. **(F)** Western blot analysis of NFATc1 and IRF8 expression in precursors of osteoclasts infected with mimics of *miR-92a-2-5p*.** (G)** Western blot analysis of NFATc1 and IRF8 expression in precursors of osteoclasts infected with or without *miR-92a-2-5p* inhibitor and treated with or without RPMI-exo. **(H)** Western blot analysis of RUNX2 expression in MSCs treated with exosomes (Health-exo, RPMI-exo or MM.1S-exo). (I) Western blot analysis of RUNX2 expression in MSCs infected with mimics of *miR-373-3p*. **(J)** Western blot analysis of RUNX2 expression in MSCs infected with or without *miR-373-3p* inhibitor and treated with or without RPMI-exo. **(K, L)** Western blot analysis of NFATc1 expression (K) in precursors of osteoclasts or RUNX2 expression (L) in MSCs treated with exosomes isolated from RPMI8226 (sh*Ctrl* and sh*A2B1*). GAPDH served as loading control. **(M)** Correlation coefficient between the levels of patient myeloma cells *miR-92a-2-5p* and *NFATc1* mRNA in myeloma patient-derived precursors of osteoclasts. **(N)** Correlation coefficient between the levels of patient myeloma cells* miR-373-3p* and *osterix* mRNA in myeloma patient-derived MSCs. The correlations were evaluated using Pearson coefficient. r, correlation coefficient. *P* value was determined by Pearson coefficient (n = 30). **(O)** Overall survival of myeloma patients with high *miR-92a-2-5p* (High) or low *miR-92a-2-5p* (Low) expression. **(P)** Overall survival of myeloma patients with high *miR-373-3p* (High) or low *miR-373-3p* (Low) expression. Data are averages ± SD. Each experiment was repeated three times. ***P < 0.001. *P* values were determined using one-way ANOVA.

We next investigated the mechanism by which exosomes miRNAs modify bone cell differentiation. We used the prediction program TargetScan to identify the potential miR-92a-2-5p or miR-373-3p targets and identified two miRNA responsive elements for miR-92a-2-5p or miR-373-3p in the 3' UTR of *IRF8* or *RUNX2* mRNAs (**Figure 6A**). We found that miR-92a-2-5p or miR-373-3p inhibited the luciferase activity of a reporter containing the wild-type *IRF8* or *RUNX2* 3' UTR but not that of a reporter with a mutated 3' UTR (**Figure 6A-B**). Real time PCR analysis and western blot revealed that monocytes NFATc1 mRNA and proteins levels are higher, IRF8 protein levels are lower treated with myeloma cells exosomes as compared to untreated cells or healthy plasma cell exosomes treated cells (**Figure 6C-D**). Similar to the exosomes treated results, monocytes transfected with miR-92a-2-5p mimics increased NFATc1 mRNA and proteins levels, inhibited IRF8 protein levels (**Figure 6E-F**). Monocytes transfected with miR-92a-2-5p inhibitor reduced miR-92a-2-5p expression (**Figure S7A**), and reversed exosomes activated NFATc1 and inhibited IRF8 expression (**Figure 6G**). Western blot analysis indicated that MSCs RUNX2 proteins levels are lower treated with myeloma cells exosomes as compared to untreated cells or healthy plasma cell exosomes treated cells (**Figure 6H**). MSCs transfected with miR-373-3p mimics inhibited RUNX2 proteins levels (**Figure 6I**). MSCs transfected with miR-373-3p inhibitor reduced miR-373-3p expression (**Figure S7B**), and reversed exosomes inhibited RUNX2 expression (**Figure 6J**). hnRNPA2B1 knockdown reduced RPMI8226 cell exosomes induced NFATc1 activation in monocytes and RUNX2 inhibition in MSCs (**Figure 6K-L**). Besides, we found a strong positive correlation between the levels of *miR-92a-2-5p* or *miR-373-3p* with *NFATc1* mRNA (**Figure 6M**), and a strong negative correlation between the levels of *miR-373-3p* with *osterix* (RUNX2 target gene) mRNA in myeloma patients (**Figure 6N**). Furthermore, we found that patients with high levels of *miR-92a-2-5p* or *miR-373-3p* in myeloma cells displayed shorter overall survival than those with low expression (**Figure 6O-P**). These experiments demonstrate that myeloma cells hnRNPA2B1 regulated monocytes IRF8 and NFATc1 or MSCs RUNX2 expression through exosomes miR-92a-2-5p or miR-373-3p.

### hnRNPA2B1 regulated exosomes miR-92a-2-5p and miR-373-3p induce bone destruction *in vivo*

**Figure 7:**
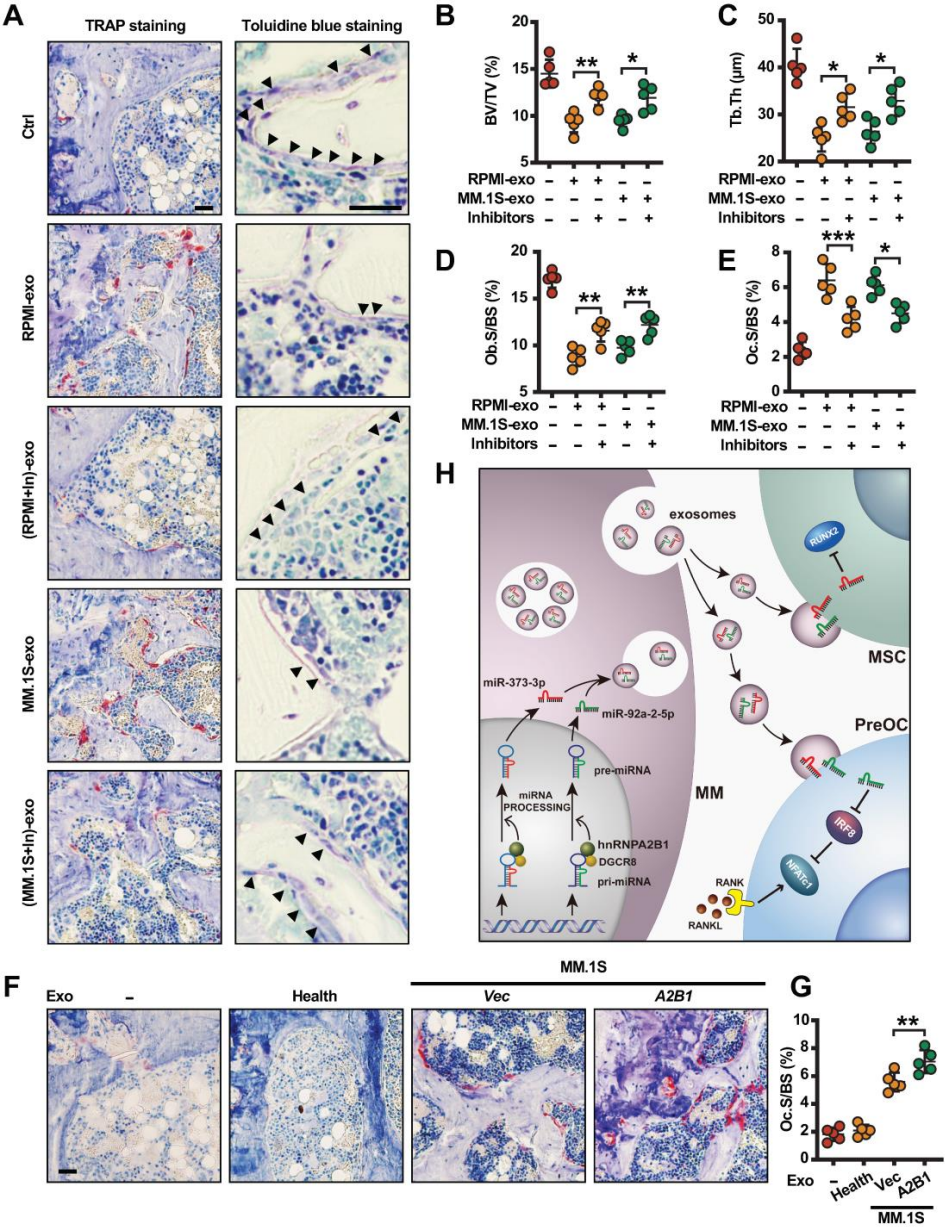
** Myeloma cell exosomes induce bone destruction *in vivo* through miR-92a-2-5p and miR-373-3p.** SCID mice femurs were injected with myeloma cell lines RPMI8226 or MM.1S isolated exosomes transfected with or without both miR-92a-2-5p and miR-373-3p inhibitors, the mice receiving PBS served as controls. After 3 weeks, mouse femurs were extracted, fixed, TRAP- or toluidine blue-stained, and analyzed. **(A)** Representative images of TRAP- or toluidine blue-stained femurs of mice. Black arrows: Osteoblast. Shown are the percentages of BV/TV **(B)**, Tb. Th **(C)**, Ob.S/BS **(D)** and Oc.S/BS **(E)**. SCID mice femurs were injected with MM.1S (*Vec* and *A2B1*) isolated exosomes, the mice receiving PBS or exosomes isolated from healthy plasma cells served as controls. Shown are the representative images of TRAP-stained femurs of mice** (F)**. and the percentage of Oc.S/BS (G). **(H)** Depiction of signaling pathways involved in the myeloma cells hnRNPA2B1-mediated suppression of osteoblastogenesis and activation of osteoclastogenesis. Data are means ± SD (n = 5 mice/group, three replicate studies). Scale bar: 50 µm. *P < 0.05; **P < 0.01; ***P < 0.001. *P* values were determined using one-way ANOVA.

Toward a therapeutic approach, we asked whether inhibiting miR-92a-2-5p and miR-373-3p can prevent myeloma-induced osteolytic bone lesions. For this purpose, we transfected RPMI8226 or MM.1S cells with miR-92a-2-5p/miR-373-3p inhibitors or control miRNAs, cultured and collected exosomes. We injected RPMI8226 or MM.1S exosomes, miR-92a-2-5p/miR-373-3p inhibitors transfected RPMI8226 or MM.1S exosomes into mouse femurs, bone samples were taken. Bone histomorphometric analysis (**Figure 7A**) showed higher percentages of BV/TV (**Figure 7B**), trabecular thickness (Tb. Th) (**Figure 7C**), Ob.S/BS (**Figure 7D**), and lower Oc.S/BS (**Figure 7E**) in the mice injected with miR-92a-2-5p /miR-373-3p inhibitors transfected myeloma cell exosomes. We also injected MM.1S (*Vec* and *A2B1*) exosomes into mouse femurs. The results showed more mature osteoclasts and higher percentages of Oc.S/BS in the mice injected with *A2B1* MM.1S exosomes compared to *Vec* MM.1S exosomes (**Figure 7F-G**). To increase translational value, we also explored whether inhibiting hnRNPA2B1 can improve bortezomib treatment effect in myeloma induced osteolytic bone disease. The results showed that, in combination with hnRNPA2B1 inhibition, bortezomib significantly reduced osteoclastogenesis or increased osteoblastogenesis than bortezomib alone (**Figure S8A-B**).

We also analyzed TCGA database and found upregulated expression of* hnRNPA2B1* in some malignancies including breast, colon, lung and liver cancer (**Figure S9A-D**). To further confirm our findings, we knocked down or over expressed hnRNPA2B1 in breast cancer cell line MCF7 (**Figure S10A**), we observed decreased growth in sh*A2B1* MCF7 cells compared with sh*Ctrl* group (**Figure S10B**), and increased growth in *A2B1* MCF7 cells compared with *Vec* group (**Figure S10B**). Similar to myeloma cells, exosomes isolated from MCF7 cells expressing high levels of hnRNPA2B1 (sh*Ctrl*, *A2B1*) induced more TRAP^+^ cells formation (**Figure S10C**) and less mature osteoblasts than those with low levels of hnRNPA2B1 (*Vec* or sh*A2B1*) (**Figure S10D**). These findings may have broader implications for the genesis of bone lesions caused by these and other tumors.

## Discussion

Osteolytic bone lesion is a hallmark in the vast majority of myeloma patients. Myeloma cells disrupt the delicate balance between bone formation and resorption, leading to debilitating osteolytic bone lesions. Although previous studies established that myeloma cell exosomes enhance osteoclast differentiation and inhibit osteoblast differentiation, the mechanism underlying remains elusive. In this study we clearly demonstrated that myeloma cells hnRNPA2B1 may be responsible, at least in part, for promoting bone destruction *in vivo* through myeloma cell exosomes. hnRNPA2B1-DGCR8 complex mediates m6A-dependent primary microRNA processing events and upregulates miR-92a-2-5p and miR-373-3p expression. These two miRNAs are packed into exosomes and transported to recipient monocytes or MSCs, leading to activating osteoclastogenesis and suppressing osteogenesis by inhibiting IRF8 or RUNX2 (**Figure 7H**). Our clinical studies examining the correlation between the level of myeloma cell hnRNPA2B1 and the number of osteolytic bone lesions in newly diagnosed patients support this conclusion. Thus, this study reveals a novel mechanism that explains how myeloma cells induce bone destruction. Our study also suggests that hnRNPA2B1 may be a therapeutic target for bone disease in patients with myeloma.

miRNAs widely found in eukaryotes, are a class of noncoding RNAs (19-25 nucleotides in length). They usually bind to the 3' UTR regions of their target mRNAs, and lead to translation inhibition or degradation 20. miRNAs play important roles in physiology and pathophysiology, including development, apoptosis, tumor development, and so on 16, 21, 22. Previous studies also showed that miRNAs are involved in osteoclastogenesis and osteoblastogenesis 23-25. More than 20 miRNAs function has been identified in osteoclast generation, and showed positive or negative feedback to regulate osteoclastogenesis. For instance, miR-340 inhibits osteoclast differentiation through repression of microphthalmia-associated transcription factor (MITF) 26, miR-29 family play a critical role in early phase of osteoclastogenesis by targeting NFIA (nuclear factor I A) 27. But the function of miR-92a-2-5p in osteoclast differentiation remains unknown. We reported that miR-92a-2-5p enhances osteoclast differentiation by inhibiting IRF8. IRF8 is a well-known transcription factor which can suppress RANKL-induced NFATc1 expression 28. In osteoblastogenesis, some studies have discovered multiple miRNAs to be important regulators of bone-forming genes, including essential transcription factors and developmental signaling molecules and their receptors that are required for the complex process of osteoblastogenesis. miR-26a negatively regulates osteoblast differentiation by targeting the SMAD family member 1 (SMAD1) 29. miRNA-133a-5p and miRNA-132-3p inhibits osteoblast differentiation by targeting the 3' UTR of *RUNX2* or E1A binding protein P300 (*EP300*) directly 30. miR-15b promotes osteoblast differentiation by protecting RUNX2 protein from SMAD specific E3 ubiquitin protein ligase 1 (Smurf1) mediated degradation 31. In our study, we found miR-373-3p binds directly to the 3' UTR of *RUNX2* and inhibits osteoblastogenesis. It is a helpful addition to the available research.

Exosomes are small membrane vesicles of endocytic origin derived from various cell types and released by fusion with the recipient cell membrane. Exosomes mediate cell-to-cell communication by transferring mRNAs, miRNAs, long non-coding RNAs (lncRNAs) and proteins. Exosomes play multiple roles in immune response, antigen presentation, tumor development, tumor metastasis, cell migration, cell differentiation, and angiogenesis, and so on 16, 17. But the effect of myeloma cell released exosomes in bone lesion remains poorly understood. A previous study suggested that myeloma cell exosomes modulate osteoclast's function and differentiation 18, but the mechanism is unknown. Another work reported that myeloma cell exosomes induced MSCs miR-103-3p expression and inhibited osteoblast differentiation 19. Our study elucidated that myeloma cells hnRNPA2B1 upregulates exosomes miR-92a-2-5p and miR-373-3p expression, which enhances osteoclastogenesis and inhibits osteoblastogenesis and thus lead to bone destruction. Furthermore, miRNA-92a-2-5p and miR-373-3p expression levels are much lower in MSCs or monocytes compared with myeloma cells. Myeloma cell packed these miRNAs and transferred to recipient cells through exosomes, which provided a new possibility of how tumor cells or other stromal cell in tumor microenvironment to mediate bone lesions.

Collectively, our results elucidate a new mechanism by which myeloma induced bone lesions. At present, lack effective targeted drug is a major barrier to myeloma bone lesion therapy. Attempts to target RANKL and DKK1 have achieved only modest success. For an example, the anti-resorptive agent denosumab (a monoclonal antibody against RANKL) only showed a moderate effect in a Phase III trial 2. BHQ880 (a monoclonal antibody against DKK-1) fails to restore new bone formation in a Phase I/II study. Bisphosphonates can suppress osteoclast function, but which are less than fully effective and cause osteonecrosis of the jaw in part of treated patients 32. Because there is no hnRNPA2B1 inhibitor in commercial, the therapeutic effect of these drugs in myeloma-induced bone lesions remains unknown. But our data make a compelling case for a role of hnRNPA2B1 in myeloma-induced bone disease and thus encourage evaluating of these inhibitors. More importantly, hnRNPA2B1 is often upregulated by other malignancies including breast, colon, liver, and lung cancer, our findings also have broader implications for the mechanisms of bone metastasis caused by these and other tumors.

## Materials and Methods

### Cell lines and primary myeloma cells

Myeloma cell lines ARP-1, H929 and U266 were provided by Dr. Zhiqiang Liu's lab of Tianjin Medical University. The MCF7 (#HTB-22), HEK293T (#ACS-4500), RPMI8226 (CCL-155), and MM.1S (CRL-2974) cells were purchased from the American Type Culture Collection. Primary myeloma cells were isolated from bone marrow aspirates of myeloma patients by using anti-CD138 antibody-coated magnetic beads (Miltenyi Biotec). Myeloma cells were maintained in RPMI1640 medium with 10% fetal bovine serum (FBS). MCF7 and HEK293T cells were cultured in Dulbecco's modified Eagle's medium (DMEM) with 10% FBS. Patient samples were obtained from the Qingdao Municipal Hospital of Qingdao University and The First Affiliated Hospital of Xiamen University. Bone lesions in the study participants were characterized by radiologists at Qingdao Municipal Hospital. This study was approved by the Ethics Committee of Xiamen University, and all protocols conformed to the Ethical Guidelines of the World Medical Association Declaration of Helsinki.

### Antibodies, plasmids, and reagents

The plasmids *hnRNPA2B1* and control vector were purchased from GeneCopoeia. MDH1-PGK-GFP 2.0 retroviral vector was purchased from Addgene (#11375). Except where specified, all chemicals were purchased from Sigma-Aldrich, and all antibodies for western blot analysis were purchased from Cell Signaling Technology. shRNAs against *hnRNPA2B1* and non-target control were purchased from Sigma-Aldrich. ELISA kits were purchased from Immunodiagnostic Systems.

### *In vitro* osteoblast and osteoclast formation and function assays

MSCs were obtained from the bone marrow, and mature osteoblasts were generated from MSCs with osteoblast differentiation medium as described previously 33. The bone formation activity of osteoblasts was determined using Alizarin red S staining (Sigma-Aldrich). Human monocytes were isolated from peripheral blood mononuclear cells and cultured to obtain the precursors of osteoclasts. The precursors derived from human monocytes were cultured in M-CSF (25 ng/ml), a low dose of RANKL (10 ng/ml) and cocultured with or without myeloma cells for 7 days to induce mature osteoclast formation. For the detection of mature osteoclasts, TRAP staining was performed using a leukocyte acid phosphatase kit (Sigma-Aldrich).

### Western blot analysis

Cells or exosomes were harvested and lysed with 1 × lysis buffer (#9803, Cell Signaling Technology). Cell lysates were subjected to SDS-PAGE, transferred to a nitrocellulose membrane, and immunoblotted with antibodies against GAPDH (#5174), hnRNPA2B1 (#9304), HSP90 (#4877), CD63 (#52090), β-actin (#3700), Calreticulin (#12238), NFATc1 (#8032), IRF8 (#83413) and RUNX2 (#12556) (Cell Signaling Technology).

### Quantitative real-time PCR of mRNAs

Total RNA was isolated using a RNeasy kit (QIAGEN). An aliquot of 1 μg of total RNA was subjected to reverse transcription (RT) with a SuperScript II RT-PCR kit (Invitrogen). Quantitative PCR was performed using SYBR Green Master Mix (Life Technologies) with the QuantStudio 3 Real-Time PCR System (Life Technologies). The reaction was performed with the following settings: 95 °C for 10 min, followed by 40 cycles of 95 °C for 15 s and 60 °C for 60 s. The primers used are listed in **Table S1**.

### Quantitative real-time PCR of miRNAs

Total RNA was isolated with the mirVana miRNA Isolation Kit (Ambion). Quantification of the mature form of miRNAs was performed with a Bulge-LoopTM miRNA Quantitative real time PCR primer kit (RiBoBio, Guangzhou, China). The U6 small nuclear RNA was used as an internal control.

### Cell viability, Cell cycle, soft agar colony formation assays and ELISA

For viability assays, cells were plated at 1 × 10^4^ cells/well in triplicate. Assays were performed using CellTiter-Glo Luminescent Cell Viability Assay Kit (Promega) or Cell Counting Kit-8 (CCK-8) (Dojindo). Cell proliferation was also measured using the 5-ethynyl-2'-deoxyuridine (EdU) assay kit (RiboBio) according to the manufacturer's instructions. For the cell cycle analysis, the cells were fixed and then stained with PI staining buffer (Multisciences Biotech) for 30 min at room temperature in the dark, and measured using flow cytometry. Soft agar colony formation assays were performed as previously described. Briefly, the 5 × 10^4^ cells in 0.4% Noble agar were plated on top of the 0.8% Noble agar bottom layer in a 6-well plate. After 3 weeks, they were stained with 1 mg/ml p-iodonitrotetrazolium chloride for visualization and counting. In addition, mouse serum was collected and measured using an ELISA kit (Immunodiagnostic Systems) according to the manufacturer's instructions.

### Immunohistochemistry

Formalin-fixed, paraffin-embedded sections of bone marrow biopsy samples obtained from patients with myeloma were deparaffinized and stained. Slides were stained with anti-CD138 (LS-B9360, LifeSpan BioSciences) and hnRNPA2B1 (LS-B10604, LifeSpan BioSciences) antibody using an EnVision System (#K5361, DAKO) following the manufacturer's instructions and counterstained with hematoxylin.

### miRNA gene cloning and ectopic expression

The human primary miR-92a-2-5p and primary miR-373-3p genes were amplified by PCR from normal genomic DNA and cloned into the MDH1-PGK-GFP 2.0 vector. The primers used in the subcloning are: pri-miR-92a-2-5p-F: 5'-ATTCTCGAGTGGGCACTTCCAGTACTCTTGGAT-3', pri-miR-92a-2-5p-R: 5'-CCGGAATTCTCGCCAACAAAGGTCCTGCGG-3'; pri-miR-373-3p-F: 5'-ATTCTCGAGACTCCAGCCTGGGCGACAGA-3'; pri-miR-373-3p-R: 5'-CCGGAA-TTCCCCGTATCCTGCCCACCCCA-3'.

### Transfection of miRNA mimics or inhibitors

Cells were transfected with the indicated miRNA mimics or inhibitors (50 nM) or control oligonucleotides (50 nM) (Thermo Fisher Scientific) using the Oligofectamine reagent (Invitrogen). At 48 hours after transfection, cells were harvested for RNA and protein analyses.

### Fluorescent Staining

MM cells were fixed with 4% formaldehyde and permeabilized with 0.3% Triton X-100 in 1 × PBS. After blocking with 2% goat serum, the cells were stained with antibodies against hnRNPA2B1 (Santa Cruz) or DGCR8 (Abcam) at 4°C overnight, followed by incubation with Alexa 594- or Alexa 488-conjugated secondary antibodies (Abclonal) for 30 min at room temperature and the cell nuclei were stained with DAPI and mounted with antifade reagent (Molecular Probes). Immunofluorescent images were acquired with an IX71 confocal microscope system (Olympus).

### Luciferase assay *in vitro*

The wild type and mutated 3' UTR of human *IRF8* and *RUNX2* were subcloned into the pGL2 vector (Addgene). Mutant forms of 3' UTR were mutated from wild type using the QuikChange site-directed mutagenesis kit (StrataGene). The constructs (2 ng) were co-transfected into HEK293T cells in 96-well plates together with 200 ng of control plasmid or plasmids expressing miR-92a-2-5p or miR-373-3p and Renilla plasmid (0.2 ng). Luciferase activity was measured 48 hours after transfection using a Dual-Luciferase Reporter Assay System (Promega) according to the manufacturer's instructions. The primers used in the subcloning are listed in **Table S2**.

### Exosome isolation and characterization

Primary myeloma cells, myeloma cell lines and breast cancer cell line were used for exosome production. In brief, cells were cultured in respective media with microvesicle-free fetal bovine serum for 48 hours. Conditioned media was collected, centrifuged twice at 3000 rpm for 10 min to remove debris. The supernatant was centrifuged at 100000 × g for 60 min to collect exosomes.

The pellet containing exosomes was resuspended in 1 × PBS buffer. They were examined by transmission electron microscopy (High Resolution Electron Microscopy Facility at Xiamen University). The hydrodynamic size distribution of exosomes were determined by dynamic light scattering (DLS) system. Purified exosomes were incubated with 4 μm-diameter aldehyde/sulphate latex beads (Interfacial Dynamics) in PBS buffer overnight at 4°C. Exosome could be stained with exosome marker antibodies, anti-CD9 (BioLegend) or anti-CD63 (BioLegend) for 30 min at 4°C, and analyzed using flow cytometer (BD Biosciences).

### Exosome uptake assay

Dil cell-labeling solution (Thermo Fisher Scientific) was used to label exosomes. Briefly, The Dil-labeled exosomes were added to the culture of precursors of osteoclasts or MSCs. After 24 h, cells were collected, fixed with 4% paraformaldehyde, stained with DAPI, and then observed under confocal microscopy.

### RNA pull-down and RIP assays

The interactions between hnRNPA2B1 and primary miR-92a-2-5p or miR-373-3p were examined using RNA pull-down assays according to the instructions of the Pierce Magnetic RNA-Protein Pull-Down Kit (Thermo Fisher Scientific). Biotinylated primary miR-92a-2-5p or miR-373-3p and antisense sequences were synthesized using a TranscriptAid T7 High Yield Transcription Kit (Thermo Fisher Scientific). The nuclear proteins obtained using a NE-PER Nuclear Protein Extraction Kit (Thermo Fisher Scientific) was incubated overnight with biotinylated primary miR-92a-2-5p or miR-373-3p, followed by precipitation with streptavidin magnetic beads. The retrieved protein was eluted from the RNA-protein complex and analyzed by immunoblotting.

The RIP assays were performed using an EZ-Magna RIP kit (Millipore). Lysates of RPMI8226 or MM.1S cells obtained using RIP lysis buffer were immunoprecipitated with RIP buffer containing anti-hnRNPA2B1 or m6A antibody-conjugated magnetic beads (Abcam). The precipitated RNAs were analyzed by Quantitative real time PCR. IgG was used as the negative control.

### *In vivo* mouse experiments, measurement of tumor burden, radiography and bone histomorphometry

CB.17 SCID mice purchased from Charles River Labs, Beijing, China, were maintained in Xiamen University Animal Care-accredited facilities. The mouse studies were approved by the Institutional Animal Care and Use Committee of Xiamen University. Myeloma cells (RPMI8226 or MM.1S) (5 × 10^5^ cell/mouse) were injected into the femurs of 8-week-old SCID mice. To monitor the tumor burden, serum samples were collected from the mice weekly and tested for myeloma-secreted M proteins using ELISA analysis. To examine the lytic bone lesions, radiographs were scanned with a Bruker In-Vivo Xtreme imaging system. Bone tissues were fixed in 10% neutral-buffered formalin and decalcified, and sections of them were stained with toluidine blue or TRAP following standard protocols. Both analyses were done using the BIOQUANT OSTEO (v18.2.6) software program (BIOQUANT Image Analysis Corporation).

### Statistical analysis

Statistical significance was analyzed using the Graphpad (Version 9.0) program with two tailed unpaired Student *t*-tests for comparison of two groups, and one-way ANOVA with Tukey's multiple comparisons test for comparison of more than two groups. *P* values less than 0.05 were considered statistically significant. All results were reproduced in at least three independent experiments.

## Supplementary Material

Supplementary figures and tables 1-2.

Supplementary table 3.

Supplementary table 4.

Supplementary table 5.
