# Supplementary material for: m6A reader hnRNPA2B1 drives multiple myeloma osteolytic bone disease
Source: Theranostics. 2022 Nov 14;12(18):7760–74. doi: 10.7150/thno.76852 (PMC9706590; doi:10.7150/thno.76852)
Supplement: Supplementary file 1 — Supplementary figures and tables 1-2. [file thnov12p7760s1.pdf]

Supplementary Figures

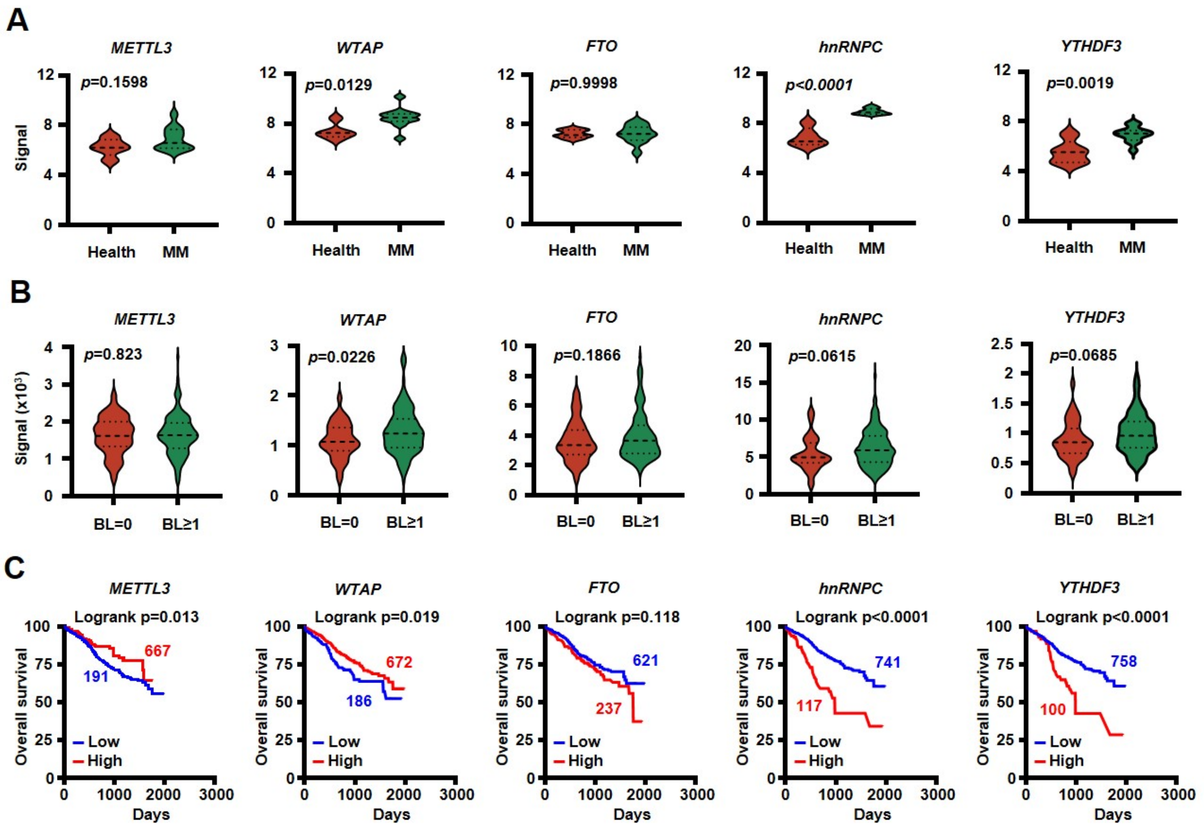

Figure S1. m6A family genes mRNA levels in GSE6691 or GSE755 datasets and analysis of survival.

(**A**) *METTL3*, *WTAP*, *FTO*, *hnRNPC* and *YTHDF3* mRNA levels in the plasma cells from myeloma patients (n = 12) compared to normal plasma cells from healthy donors (n = 5) (GEO: GSE6691). (**B**) *METTL3*, *WTAP*, *FTO*, *hnRNPC* and *YTHDF3* mRNA levels in malignant plasma cells of 37 myeloma patients without bone lesion (BL = 0) and 136 myeloma patients with bone lesion (BL ≥ 1) (GEO: GSE755). Data shown as averages ± SD. *P* values were determined by Student's *t* test. (**C**) Overall survivals in myeloma patients with high or low *METTL3*, *WTAP, FTO*, *hnRNPC* or *YTHDF3* expression.

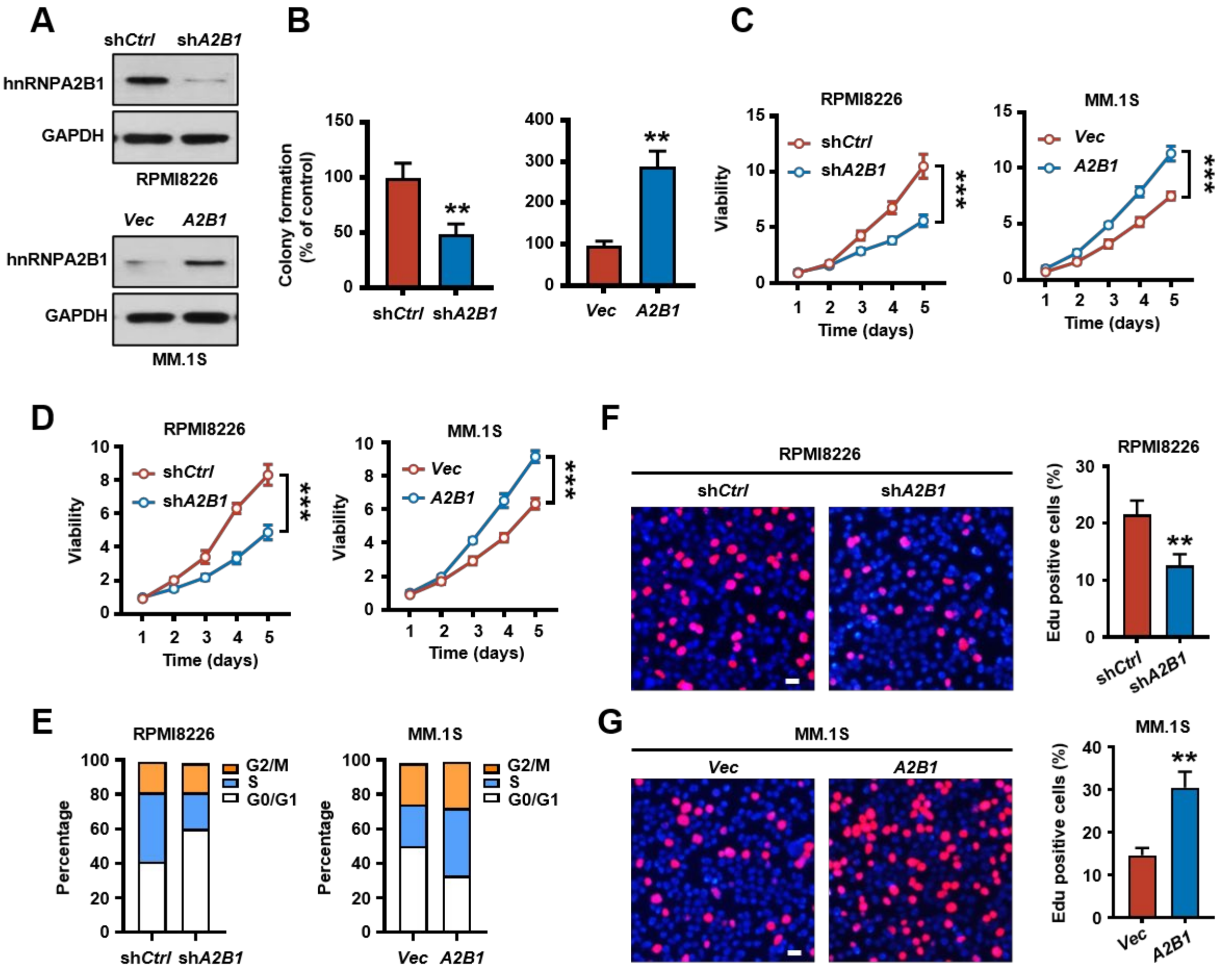

Figure S2. hnRNPA2B1 is associated with the growth of myeloma cells.

(**A**) Western blot shows expression of hnRNPA2B1in RPMI8226 cells transfected with non-targeted shRNA (sh*Ctrl*) or *hnRNPA2B1* shRNA (sh*A2B1*). MM.1S transfected with *hnRNPA2B1* cDNA (*A2B1*) or control vector (*Vec*). GAPDH served as western blot analysis loading control. (**B**) Summarized data for relative colony formation (colonies formed in sh*Ctrl* or *Vec* cells set to 100%). *P* values were determined by Student's *t* test. (**C**) Proliferation of RPMI8226 cells (sh*Ctrl* or sh*A2B1*) or MM.1S cells (*Vec* or *A2B1*) in culture for 4 days, as determined by CellTiter-Glo Luminescent Cell Viability Assay. (**D**) CCK-8 assay showed the proliferation of RPMI8226 cells (sh*Ctrl* or sh*A2B1*) or MM.1S cells (*Vec* or *A2B1*) in culture for 4 days. (**E**) Cell cycle analysis was performed with flow cytometry in RPMI8226 cells (sh*Ctrl* or sh*A2B1*) or MM.1S cells (*Vec* or *A2B1*). *P* values were determined using one-way ANOVA. (**F, G**) Representative images and the percentage of EdU-positive cells of RPMI8226 cells (sh*Ctrl* or sh*A2B1*) or MM.1S cells (*Vec* or *A2B1*), as determined by EdU staining assay. Scale bar, 10 μm.

P values were determined by Student's *t* test. Data are averages ± SD. Each experiment was repeated three times. $^{**}P < 0.01$; $^{***}P < 0.001$.

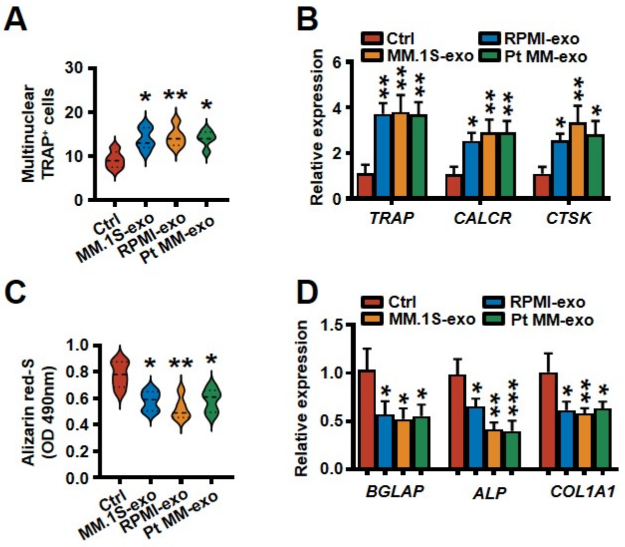

Figure S3. Myeloma cells exosomes enhance osteoclast differentiation and inhibit osteoblast differentiation *in vitro*.

Precursors of osteoclasts were cultured in osteoclast medium treated with exosomes (20 µg/ml) isolated from MM.1S culture medium (MM.1S-exo), RPMI8226 culture medium (RPMI-exo) or patient myeloma cells culture medium (Pt MM-exo). Shown are the numbers of multinuclear (≥ 3) TRAP$^+$ cells (**A**) and relative expression of the *TRAP*, *CALCR*, and *CTSK* genes (**B**). MSCs were cultured in osteoblast medium treated with MM.1S-exo, RPMI-exo or Pt MM-exo (20 µg/ml). Shown are the summarized data of Alizarin red S staining (**C**) and the relative expression of *BGLAP*, *ALP*, and *COL1A1* genes (**D**). Addition of PBS served as a control. Data are averages ± SD. Each experiment was repeated three times. **P* < 0.05; ***P* < 0.01; ****P* < 0.001. All *P* values were determined using one-way ANOVA.

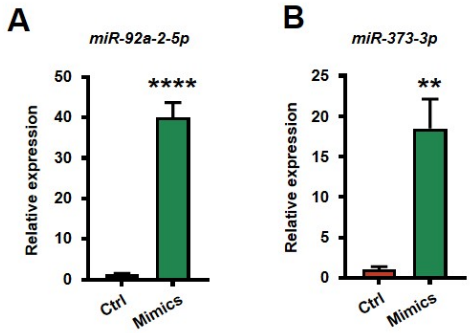

Figure S4. miR-92a-2-5p and miR-373-3p expression levels in monocytes or MSC transfected with miRNA mimics.

Quantitative real-time PCR analysis shows the relative expression of *miR-92a-2-5p* (**A**) or *miR-373-3p* (**B**) in precursors of osteoclasts or MSCs transfected with *miR-92a-2-5p* or *miR-373-3p* mimics. Data are averages ± SD. Each experiment was repeated three times. ***P* < 0.01; *****P* < 0.0001. *P* values were determined by Student's *t* test.

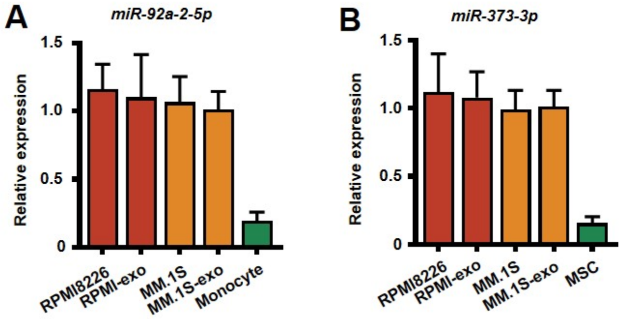

Figure S5. miR-92a-2-5p and miR-373-3p expression levels in myeloma cells, exosomes, monocytes and MSCs.

Quantitative real-time PCR analysis shows the relative expression of *miR-92a-2-5p* (**A**) or *miR-373-3p* (**B**) in myeloma cells (RPMI8226, MM.1S), myeloma cells exosomes (RPMI-exo, MM.1S-exo), monocytes or MSCs. Data are averages ± SD. Each experiment was repeated three times.

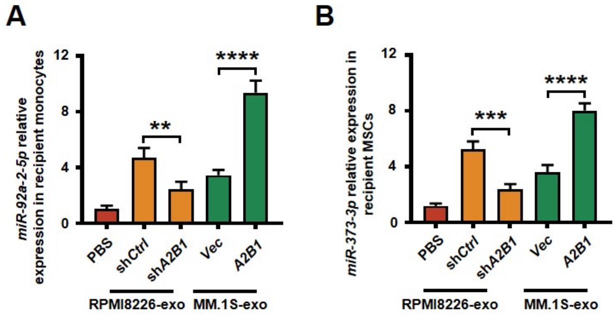

Figure S6. miR-92a-2-5p and miR-373-3p are packaged into exosomes and transported to recipient monocytes or MSCs.

Quantitative real-time PCR analysis shows the relative expression of *miR-92a-2-5p* (**A**) or *miR-373-3p* (**B**) in recipient cells treated with exosomes isolated from RPMI8226 (sh*Ctrl*, sh*A2B1*) and MM.1S (*Vec*, *A2B1*). Data are averages ± SD. Each experiment was repeated three times. **$P < 0.01$; ***$P < 0.001$; ****$P < 0.0001$. All P values were determined using one-way ANOVA.

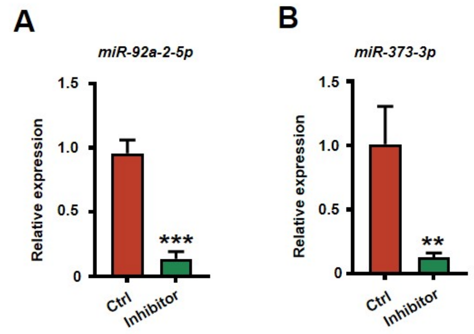

Figure S7. miR-92a-2-5p and miR-373-3p expression levels in monocytes or MSCs transfected with miRNA inhibitors.

Quantitative real-time PCR analysis shows the relative expression of *miR-92a-2-5p* (**A**) or *miR-373-3p* (**B**) in precursors of osteoclasts or MSCs transfected with *miR-92a-2-5p* or *miR-373-3p* inhibitors. Data are averages ± SD. Each experiment was repeated three times. $^{**}P < 0.01$; $^{***}P < 0.001$. *P* values were determined by Student's *t* test.

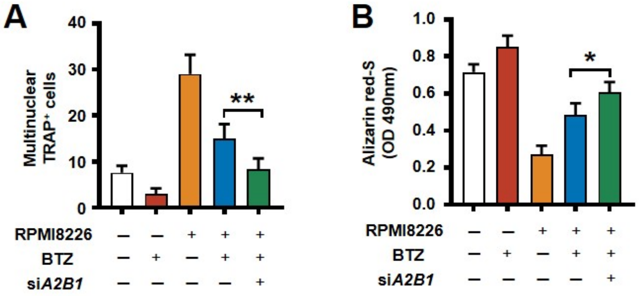

Figure S8. Knockdown of hnRNPA2B1 promotes bortezomib efficiency in controlling myeloma-associated osteoclastogenesis activation and osteoblastogenesis inhibition. Precursors of osteoclasts or MSCs were co-cultured with RPMI8226 cells transfected with or without siRNA against *hnRNPA2B1* (si*A2B1*) in the presence of bortezomib (10 nM) or not. Shown are the numbers of multinuclear (≥ 3) $TRAP^{+}$ cells (**A**) and summarized data of Alizarin red S staining (**B**). Data are averages ± SD. Each experiment was repeated three times. $*P < 0.05$; $**P < 0.01$. All *P* values were determined using one-way ANOVA.

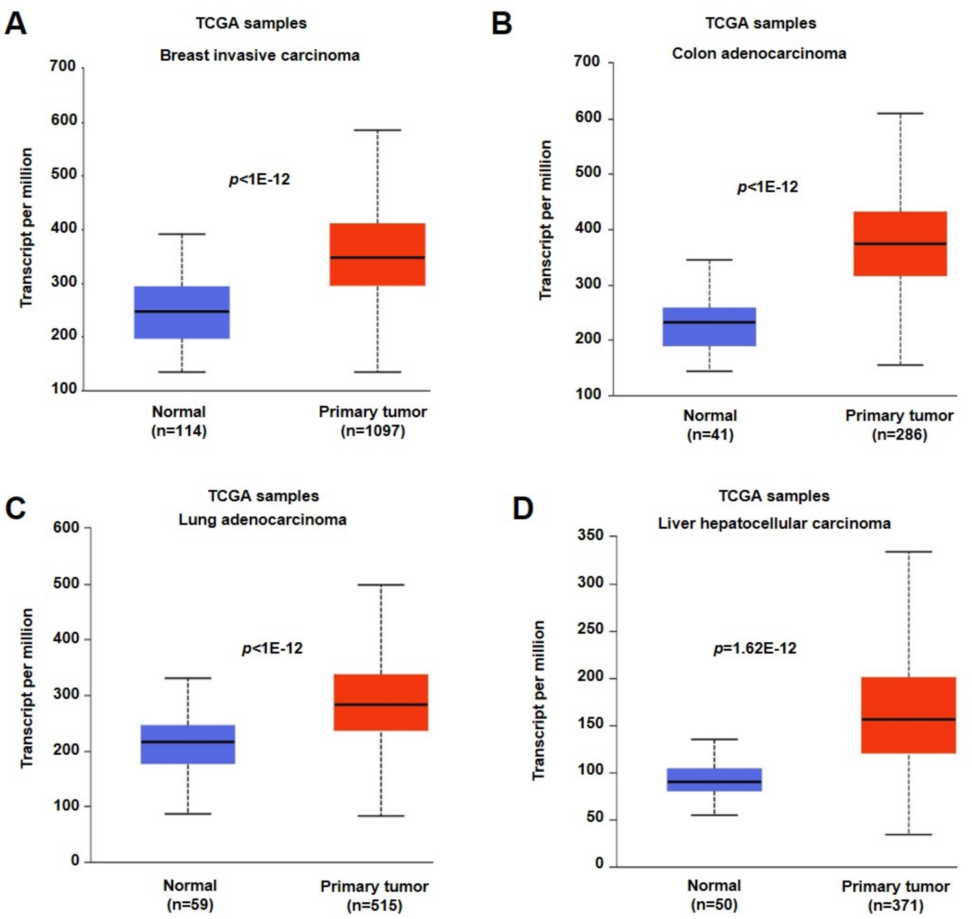

Figure S9. Expression of hnRNPA2B1 is elevated in some types of solid tumors. Analysis of TCGA data assessing the *hnRNPA2B1* mRNA gene expression of in breast cancer cells (**A**), colon cancer cells (**B**), lung cancer cells (**C**) and liver cancer cells (**D**) compared with normal cells. Data are represented as mean ± SD. *P* values were determined by Student's *t* test.

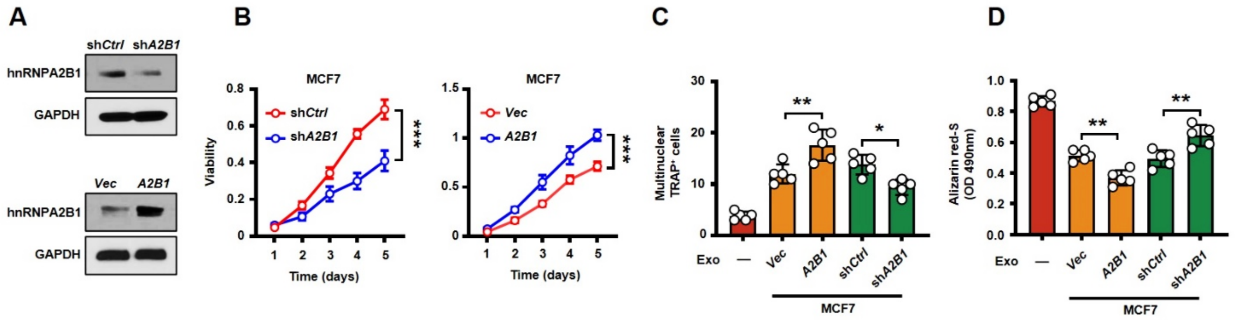

Figure S10. Breast cancer cells hnRNPA2B1 promote tumor cell growth, enhance osteoclastogenesis or inhibit osteoblastogenesis via exosomes.

(**A**) Western blot shows expression of hnRNPA2B1 in MCF7 cells transfected with non-targeted shRNA (sh*Ctrl*) or *hnRNPA2B1* shRNA (sh*A2B1*). MCF7 transfected with *hnRNPA2B1* cDNA (*A2B1*) or control vector (*Vec*). GAPDH served as loading control. (**B**) Proliferation of MCF7 (*Vec* and *A2B1*) or MCF7 (sh*Ctrl* and sh*A2B1*) cells in culture for 4 days. Precursors of osteoclasts or MSCs were cultured in osteoclast medium or osteoblast medium treated with exosomes (20 µg/ml) isolated from MCF7 (*Vec* and *A2B1*) or MCF7 (sh*Ctrl* and sh*A2B1*) cells culture medium. Shown are numbers of multinuclear (≥ 3) $TRAP^+$ cells (**C**) and summarized data of Alizarin red S staining (**D**). Addition of PBS served as control. Data are averages ± SD. Each experiment was repeated three times. **P* < 0.05; ***P* < 0.01; ****P* < 0.001. All *P* values were determined using one-way ANOVA.

Supplementary tables

Table 1. Primers used in real time reverse transcription PCR analysis

Gene	Forward	Reverse
GAPDH	CTGGGCTACACTGAGCACC	AAGTGGTCGTTGAGGGCAATG
hnRNPA2B1	ATTGATGGGAGAGTAGTTGAGCC	AATTCCGCCAACAAACAGCTT
TRAP	AGATCCTGGGTGCAGACTTC	AAGGGAGCGGTCAGAGAATA
CALCR	GGGAATCCAGTTTGTCGTCT	ACAAAGAAGCCCTGGAAATG
CTSK	CCATATGTGGGACAGGAAGA	CCTCTTCAGGGCTTTCTCAT
BGLAP	ACTGTGACGAGTTGGCTGAC	AAGAGGAAAGAAGGGTGCCT
ALP	TCCCAGTTGAGGAGGAGAAC	CCCAGGAAGATGATGAGGTT
COL1A1	TGTTCAGCTTTGTGGACCTC	GGTGATTGGTGGGATGTCTT
RUNX2	TCAACGATCTGAGATTTGTGGG	GGGGAGGATTTGTGAAGACGG
NFATc1	CACCGCATCACAGGGAAGAC	GCACAGTCAATGACGGCTC

Table 2. **Luciferase assay primers.**

Name	**Forward**	**Reverse**
pGL2-IRF8	CATCTCGAGCCAGGTCTTCCGGATGTTTCCAG	CAGAAGCTTCACCGACATCTCGGCAGGGC
pGL2-IRF8-Mut	GATGGATGCAGGACGCAGACGGCCGTTAACGCCCAAGCGACGCACTTAGAC	GTCTAAGTGCGTCGCTTGGGCGTTAACGGCCGTCTGCGTCCTGCATCCATC
pGL2-RUNX2	CATCTCGAGAGCTTGAAGCACACCACTGTCCA	CAGAAGCTTTGGTTGGAGTGAGGGTGGAGGG
pGL2-RUNX2-Mut	AAATGTGTAACCAGACACTGGCTTTTTAAGGTAGGCTGAAACAAACACACATATTTTACACTTAC	GTAAGTGTAAAATATGTGTGTTTGTTTCAGCCTACCTTAAAAAGCCAGTGTCTGGTTACACATTT
